# Seed Oil Contents, Fatty Acid Compositions, and Gossypol Concentrations of Some Okra Landraces

**DOI:** 10.1002/fsn3.4535

**Published:** 2024-10-28

**Authors:** Faik Kantar, Mehmet Fatih Cengiz, Sabri Erbaş, Ümit Babacan

**Affiliations:** ^1^ Department of Agricultural Biotechnology, Faculty of Agriculture Akdeniz University Antalya Türkiye; ^2^ Department of Field Crops, Faculty of Agriculture Isparta University of Applied Sciences Isparta Türkiye

**Keywords:** *Abelmoschus esculentus*, fatty acids, gossypol, okra, seed oil

## Abstract

Okra has recently attracted attention owing to its superior tolerance to high temperatures, greater adaptation to poor soil conditions, and having a robust plant structure. The plant contains a high amount of oil and valuable fatty acids; however, the main restriction of using okra seeds as an oil crop results from its gossypol contents. The aim of this study was to determine the oil content of okra landraces and to evaluate its potential as an oil crop. For this aim, seed oil content, fatty acid compositions of cold‐pressed seed oil, and gossypol concentrations of fruit, oil cake, and seed oil were investigated in a core collection of 26 okra landraces, lines, and cultivars. Individual plants were harvested at the full maturity stage, and seeds were harvested and dried under 35°C for 2 days prior to oil extraction. Oil content, fatty acid composition, and gossypol content were analyzed by NMR, GC‐FID, and HPLC, respectively. The calibration coefficients (*r* ^*2*^) of all the methods were determined to be > 0.99. The seed oil content of the samples ranged between 12.15% and 18.83%. Linoleic (42.01%), palmitic (31.65%), oleic (18.39%), and stearic acids (3.20%) were found to be the largest fraction of the fatty acids. The data matrix from 19 fatty acids and oil content was subjected to Principle Component Analysis (PCA). As a result, 6 principal components (PCs, eigenvalues > 1) explained 83.84% of total variance in the data set, with PC1 contributing 32.69% of the total. Gossypol contents of the fruit, oil cake, and seed oil fractions ranged between LOQ‐2.12, < LOQ‐7.01, and < LOQ‐62.46 mg/kg, respectively. In conclusion, okra may have the potential to be an alternative oil crop for food/feed purposes due to the presence of reasonable oil content, high‐quality fatty acid variations, and very low amounts of toxic gossypols, warranting further breeding and agronomic studies.

## Graphical abstract

The okra plant contains a high amount of oil and valuable fatty acids; however, the main restriction to the use of okra seed as an oil crop results from its gossypol contents. This work attempted to investigate the oil content, oil composition, and gossypol content in fruits, oil cake, and plant oil in the germplasm of cultivars and landraces. The results highlight the importance of okra as an alternative oil crop for the areas under the influence of increasing warming temperatures.

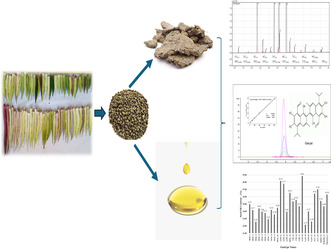

## 1 Background

Edible plant oil is a crucial nutritional resource for human health. Demand for edible plant oil has been rising day by day as the global population grows, and many oil‐bearing plants are being grown on a large scale extensively using agricultural technologies and biotechnological methods, such as genome editing, cell engineering, and tissue culture. In addition, with the improvement of agricultural processing technologies, more plants have been introduced for edible oil production. Okra (*Abelmoschus esculentus* (L.) Moench), which belongs to the Malvaceae family, is a warm‐temperature crop widely grown in the tropics and sub‐tropics from Africa to Asia, Europe, and America, with its geographical distribution reaching up to latitudes of 35–40 (Akinyele and Temikotan 2007).

Okra is commonly grown for its vitamins, water‐soluble polysaccharides, bioactive compounds, antioxidants, and mineral‐rich fresh and dry fruits (Gemede et al. 2018; Guo et al. 2024; Olawuyi and Lee 2021; Wang et al. 2020; Yasin et al. 2020). It is a multipurpose crop with a variety of uses. Okra seeds are processed for use as coffee‐like beverages (Calisir et al. 2005). The seeds can be used to increase the amount of phenolics in wheat bread (Xu et al. 2020). Seed meal is used in probiotic yogurt making (Liu, Xie, and Nie 2020). Although okra is not grown as an oil crop in the world, previous studies show that okra seeds contain 13.0% and 38.1% edible oil (Anwar et al. 2010; Martin and Rhodes 1983), which is comparable to soybean (17.0%–21.0%), cotton seed (15.0%–24.0%), and safflower (25.0%–40.0%) (Sikorski and Kolakowska 2010). Okra oil had certain similarities with cottonseed oil such as being a good source of unsaturated linoleic acid contents (32.22%–43.07%) (Liu et al. 2019).

Potential restrictions for the use of okra seed as an oil crop include the contents of toxic gossypol or gossypol‐like compounds (Camciuc et al. 1998). Gossypol, a polyphenolic dimer sesquiterpene derivative, is an antinutritional metabolite with detrimental effects on humans and monogastric animals, ruminants, and fish (Blom et al. 2001; Henry, Pesti, and Brown 2001). Dry cottonseeds (*Gossypium hirsutum*) had 8470 mg/kg gossypol, which restricted its use as oilcake and oil source (Sotelo et al. 2005). A maximum amount of 7000 mg/kg in cottonseed products is a permitted level of gossypol for use in animal feeding stuff in EU (2002b). In addition, gossypol levels as low as 0.12 and 0.35 mg/kg b.w. inhibited spermatogenesis in humans and monkeys (EFSA 2008). On the other hand, the US Food and Drug Administration (FDA) recommended the free gossypol level as 450 mg/kg for direct supplement to food human consumption not exceed (FDA 2020). Okra seeds are known to contain gossypol. Gossypol‐like compounds in okra seeds ranged between 14 and 55 mg/kg (Martin and Rhodes 1983).

Increases in the earth's temperatures associated with climate change put pressure on mainstream agriculture, which is reported to be the case in the southern parts of Africa (Ndhlala and Ngobese 2022). Okra is regarded as one of the underutilized crop species in the face of climate change (Mkhabela et al. 2022). As search for novel crops and cultivars is an important priority on the breeding programs, okra is attracting attention to serve as a potential crop with favorable characteristics (Akanbi et al. 2009; Atijegbe, Nuga, and Lale 2014). Okra is a highly resilient warm‐season crop with tolerance to high temperatures and a better adaptation to poor soil conditions. The plant contains a high amount of oil and valuable fatty acids. However, the main restriction to the use of okra seed as an oil crop results from its gossypol contents. Although some permitted values by EFSA and FDA for gossypol contents in cotton seed to use as food and feed purposes, there have been no declared standards for gossypol contents in okra samples. Therefore, further studies are needed on variation in germplasms for seed oil contents, fatty acid compositions, and gossypol concentrations in order to launch breeding programs.

This study evaluated a core germplasm for seed oil content, fatty acid variations, and gossypol contents in fruits, oil cake, and seed oil that is expected to lay a starting base for breeding oil seed okra cultivars to generate interest in okra as a potential oil crop that may be exploited for warm conditions.

## 2 Materials and Methods

### 2.1 Seed Materials

Seed material of 26 okra *Abelmoschus esculentus* (L.), as presented in Table 1, inbred single seed progenies from an ongoing breeding work from landraces and cultivars (Kantar et al. 2021), was investigated for seed oil content, fatty acid composition, and gossypol content in this study. The germplasm included 20 okra landraces (BLK‐1, MGL‐2, MGL‐3, MGL‐4, MGL‐5, MGL‐6, MGL‐7, MGL‐8, MGL‐9, MGL‐10, AYD‐11, AYD‐12, AYD‐13, MGL‐14, UIS‐15, UIS‐16, USK‐17, AYD‐18, GAN‐19, and GAN‐21), two breeders advanced lines (YLV‐22 and YLV‐23), and 3 commercially registered cultivars (Akköy‐41, Kabaklı‐11, and Marmara‐1), which were obtained from Ataturk Central Horticultural Research Institute (Yalova, Türkiye). One commercial standard cultivar (STD‐20) was purchased from the market.

**TABLE 1:** Okra landraces, lines, and cultivars investigated in the experiment.

No	Landraces no/name	Geographical location	Source^a^	Name
1	BLK‐1	Balıkesir	PC	Landrace
2	MGL‐2	Muğla	PC	Landrace
3	MGL‐3	Muğla	PC	Local white
4	MGL‐4	Muğla	PC	Local red
5	MGL‐5	Muğla	PC	Local yellow
6	MGL‐6	Muğla	PC	Local mix
7	MGL‐7	Muğla	PC	Landrace
8	MGL‐8	Muğla	PC	Landrace
9	MGL‐9	Muğla	PC	Local red
10	MGL‐10	Muğla	PC	Landrace
11	AYD‐11	Aydın	PC	Landrace
12	AYD‐12	Aydın	PC	Landrace
13	AYD‐13	Aydın	PC	Landrace
14	MGL‐14	Muğla	PC	Endeze
15	UIS‐15	Muğla	PC	Landrace
16	UIS‐16	Muğla	PC	Landrace
17	USK‐17	Uşak	PC	Sultani
18	AYD‐18	Aydın	PC	Tasbatan
19	GAN‐19	Gaziantep	PC	Landrace
20	STD‐20	Konya	ST	Sultani
21	GAN‐21	Gaziantep	PC	Landrace
22	YLV‐22	Yalova	ACHRS	BAL
23	YLV‐23	Yalova	ACHRS	BAL
24	Akköy‐41	Yalova	ACHRS	Cultivar
25	Kabaklı‐11	Yalova	ACHRS	Cultivar
26	Marmara 1	Yalova	ACHRS	Cultivar

^a^PC = Personal Collection; BAL = Breeders Advanced Line; ACHRS = Ataturk Central Horticultural Research Institute, Yalova, Turkiye.

### 2.2 Plant Materials

Plant materials were produced under isolation at an experimental farm located in Akdeniz University, Faculty of Agriculture (Türkiye). For this aim, seeds from each genotype were sown in the soil in 6 m rows with 1 m between and 20 cm within row spacing in an augmented statistical design under greenhouse conditions. The soil had a high lime content (17.70%), clay loam texture with a slightly alkali pH of 7.62, good organic matter content (2.10%), high Ca content (0.40%), low P content (0.01%), total *N* content of 0.09%, high *K* content (0.19%), and optimum Mg content (0.09%). The minor elements of the soil (Mn, Zn, Cu, and Fe) were determined to be 2.67, 0.47, 0.25, and 1.20 mg/kg respectively. Drip irrigation was employed as required, with regular fertilizer application (Düzyaman and Vural 2003).

### 2.3 Chemicals

Gossypol and fatty acid analytical standards were obtained from Sigma‐Aldrich (St. Louis, USA). The content of fatty acids analytical standard (Sigma, Supelco 37 Component FAME Mix) was C14:0 = miristic; C15:0 = pentadecenoic; C15:1 = cis‐10‐pentadecenoic; C16:0 = palmitic; C16:1 = palmitoleic; C17:0 = heptadecenoic; C17:1 = cis‐10‐heptadecenoic; C18:0 = stearic; C18:1n9t = elaidic; C18:1n9c = oleic; C18:2n6t = linolelaidic; C18:2n6c = linoleic; C20:0 = arachidic; C20:1c1n9 = cis‐11‐eicosenoic; C18:3n3 = linolenic; C20:2c11 = cis‐11,14‐eicosadienoic; C20:4n6 = arachidonic; C22:2 = cis‐13,16‐docosadienoic; and C24:0 = lignoceric.

All chemicals, with the exception of analytical reference standards, were of liquid chromatographic grade. Acetic acid, methanol, n‐hexane, acetonitrile, acetone, and phosphoric acid (H_3_PO_4_) were purchased from Isolab (Wertheim, Germany). Stock solutions of analytical reference standards were prepared in methanol. The water for preparation of the mobile phase was provided by an ultrapure water purification system of MP Minipure (Ankara, Türkiye).

### 2.4 Sample Preparation

Fruit samples for gossypol analysis were taken at the fruit set stage from the plants and air‐dried at 25°C and 65% relative humidity for 7 days. Individual plants were also harvested at the full maturity stage, and seeds were harvested and dried under 35°C for 2 days. Dried seeds were subjected to cold‐press extraction to obtain seed oil and oil cake. Oil content, fatty acid composition, and gossypol concentrations were determined in the cold‐pressed extracted oil. Gossypol concentration was performed also in fruit and oil cake.

### 2.5 Seed Oil Content and Fatty Acid Composition Analysis

Seed oil contents of cold‐pressed okra seeds were determined by Nuclear Magnetic Resonance (NMR). The oil contents of the okra seed genotypes with low, medium, and high oil content were analyzed and the oil contents were determined, and these genotypes were read in the NMR and a calibration graph was created (*r* ^*2*^ = 0.992). All genotypes were then analyzed.

To determine the fatty acid compositions, the obtained cold‐pressed oil was converted to fatty acid methyl esters (FAME) (Marquard 1987). According to the method, the extracted oil was homogenized with n‐hexane. The reactive mixture of Na‐methoxide (0.5 g), n‐hexane (20 mL), and methanol (80 mL) were added to the oil (25 μL). The prepared sample waited overnight, and 1000 μL of n‐hexane was added to the mixture. Prepared FAME was injected at a volume of 1.0 μL and determined in a gas chromatography with a flame ionization detector (GC‐FID). Analytical conditions were performed as follows: injector temperature: 250°C; nitrogen flow rate: 40 mL/min; detector temperature: 250°C; oven temperature: 140°C for 10 min and to 240°C at a rate of 3°C/min and then constant at 240°C for 10 min; total running time: 65 min; carrier gas: nitrogen; and split ratio: 120. FAME determination was performed by comparing the relative retention times with a commercial standard mix of FAME.

### 2.6 Gossypol Analysis

The content of gossypol in the samples was determined by high‐performance liquid chromatography (HPLC) equipped with a diode array detector (DAD). For sample preparation, dried fruit and oil cake samples were powdered to acquire fine powder by a laboratory‐type grinder and sieved with a 150‐μm sieve. The powdered samples were stored in a 50‐mL centrifuge tube at room temperature. Additionally, cold‐pressed extracted oil samples were also analyzed for the gossypol content. These samples (dried okra fruits, cold‐pressed okra oils, and oil cakes) were analyzed separately.

The gossypol extraction method was performed with minor modifications according to the method reported by Aoyama (2008). For this purpose, 0.2 g of ground or oil sample (for a solid sample) was put into a beaker and 10 mL of reactant (84 mL of acetic acid, 1 mL of phosphoric acid, and 15 mL of ultrapure water). The beaker was transferred to an ultrasonic process bath for 30 min at room temperature. At the end of the ultrasonic process, the obtained solution was filtered through Whatman No. 1. The system's frequency of the ultrasonic process was 37 kHz, and irradiation power (120 W) was kept constant at 100% during all treatments. Three milliliter of the aqueous layer was filtered through a 0.45‐μm syringe filter. 0.2 mL of purified solution was diluted with 1 mL of dilution solvent (50% acetone and 50% ultrapure water) in an HPLC vial and 20 μL of the obtained final solution was injected into HPLC for the determination of gossypol contents. Chromatographic conditions of the HPLC system are presented in Table 2.

**TABLE 2:** HPLC system analytical conditions.

Device	Agilent 1200 series
Detector	Diode Array Detector (DAD)
Column	C18 column (4.6 × 250 mm) 5 μm
Flow rate	1.5 mL/min
Column temperature	40°C
Injection volume	20 μL
Wavelength	254 nm
Mobile phase	90% Methanol in ultrapure water (pH level was 2.6)
Mobile phase profile	%65 A, %35 B (isocratic)
Total run	20 min

### 2.7 Method Validation of Gossypol Analysis

**FIGURE 1:**
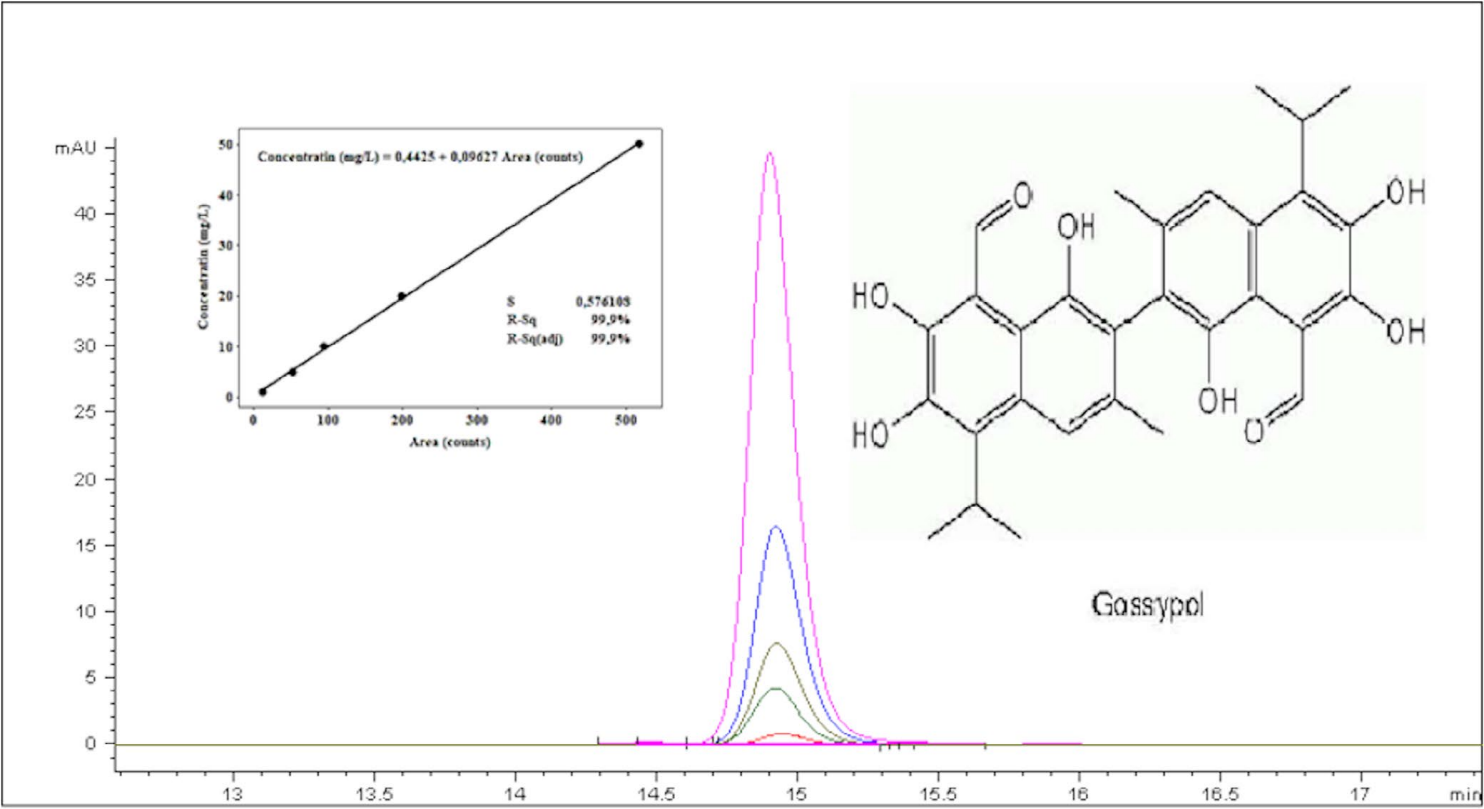
A sample HPLC chromatogram of gossypol.

A series of analytical standards for gossypol (1–500 mg/L) were prepared and injected into the HPLC, and the areas and retention times of relevant peaks were determined. A five‐point calibration curve was plotted for the concentrations, and *r* ^*2*^ was obtained as 0.999. In order to determine the limit of detection and limit of quantification (LOD and LOQ) values, the lowest concentration of analysis solutions used in the calibration line was injected into the HPLC device 10 times, and the standard deviation (SD) of the obtained peak areas was calculated. Three times the standard deviation value is specified as the LOD and 10 times the LOQ. The repeatability value was obtained by six injections with analytical standards. The relative standard deviation value of the results was used to determine the repeatability. A sample chromatogram at different concentrations of gossypol and a calibration curve of gossypol are presented in Figure 1. LOD, LOQ, and repeatability values of the gossypol analysis were determined to be 0.080 mg/L, 0.265 mg/L, and 1.825%, respectively. Based on these determined data, this method was found to be linear for the concentration levels expected from the samples, sufficient for the LOD, LOQ, and repeatability values.

### 2.8 Data Analysis

The data produced were subjected to statistical analysis using Excel 2010, Minitab 17, and the SPSS version 22 statistical package program. Principle component analysis (PCA) was performed on oil content and fatty acid composition data were determined. The similarity index was analyzed, and a distance cluster dendrogram was produced using the SPSS version 22 software package with the Euclidian distance interval and between‐groups linkage cluster method. The eigenvalues of oil content and fatty acid data were determined based on the similarity coefficient and variation.

## 3 Results and Discussion

### 3.1 Oil Contents

**FIGURE 2:**
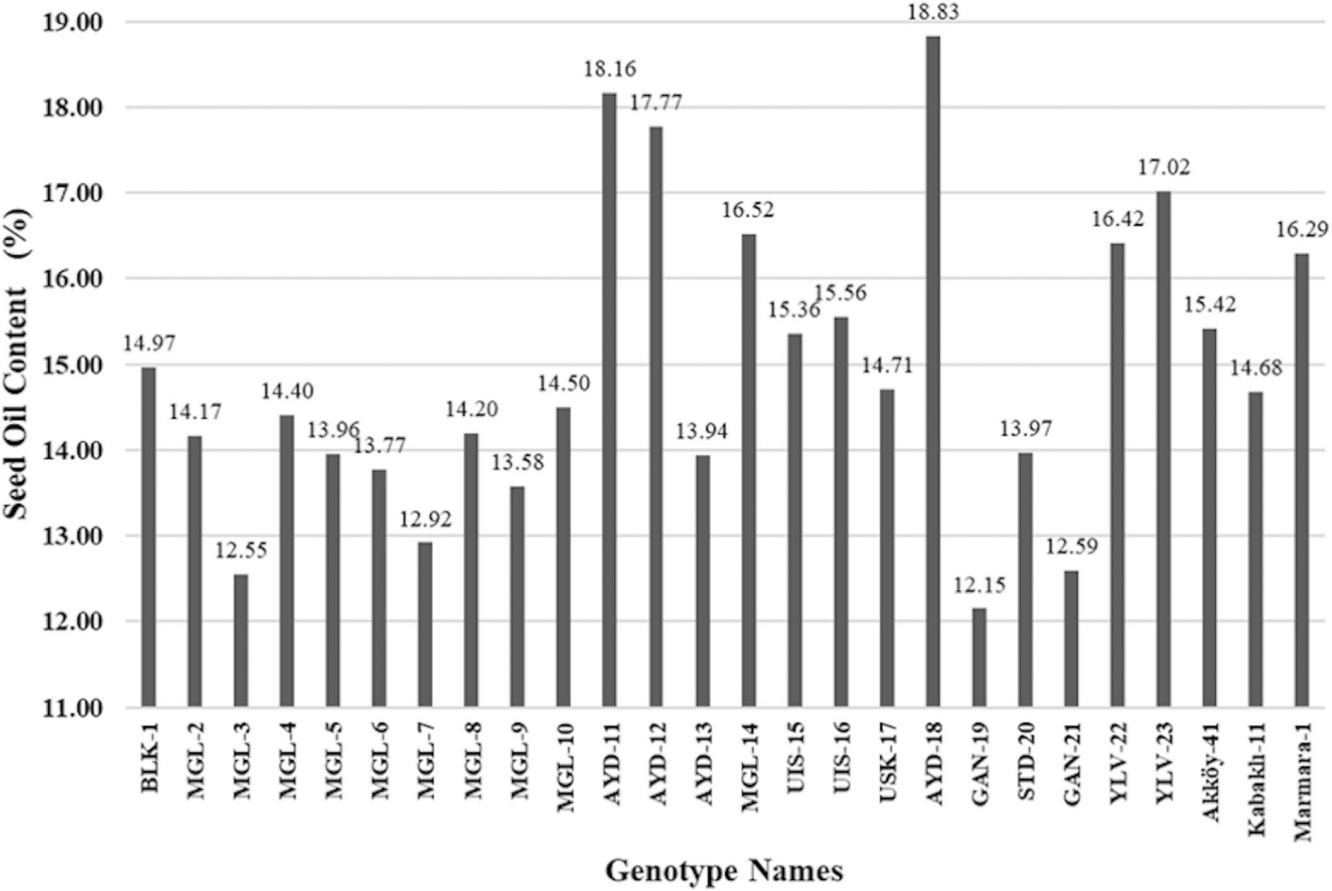
Seed oil content (%) of okra genotypes investigated.

The seed oil content of okra genotypes ranged between 12.15% and 18.83% with an average of 14.90% (Figure 2). Marmara 1, AYD‐11, YLV‐22, YLV‐23, MGL‐14, AYD‐18, and AYD‐12 had relatively high seed oil content (over 16.20%–18.83%).

Determined oil content values in the study were within the range of previous reports for okra (8%–22%) (Anwar et al. 2010; Calisir et al. 2005; Gemede et al. 2015; Martin 1982; Martin and Rhodes 1983; Rao 1985). An exceptionally high value of 40% reported by (Martin 1982) was analyzed on a sample that excluded seed coats. High oil contents of 18% in the study can be compared with the amounts commonly found in cotton seed (15.0%–24.0%) and soybean (17.0%–21.0%) (Knothe and Steidley 2005; Sikorski and Kolakowska 2010).

Okra is mainly grown for its fruits as a vegetable crop in Türkiye (FAO 2019). Normally, the plant is not regarded as an oil crop. Since demand for edible oils is steadily increasing and the search for new oilseed crops continues, okra may be considered as an alternative oil crop (Akanbi et al. 2009; Atijegbe, Nuga, and Lale 2014).

### 3.2 Fatty Acid Compositions

**FIGURE 3:**
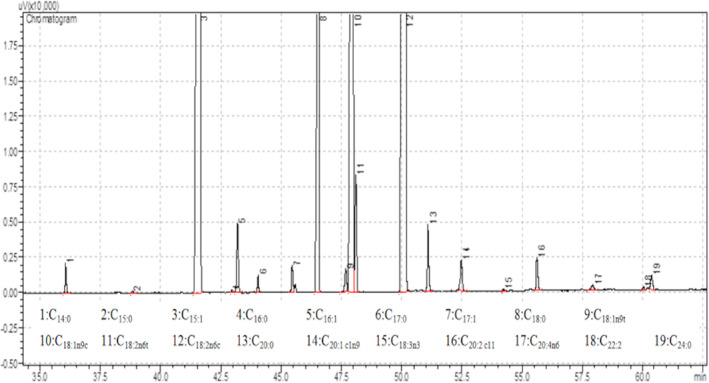
A representative chromatogram of fatty acid profiles of cold‐pressed oil specimen from genotype AYD‐18.

A representative GC‐FID chromatogram of the determined fatty acid methyl esters from okra seed oil is presented in Figure 3. A total of 19 fatty acids (C14:0 = Miristic; C15:0 = Pentadecenoic; C15:1 = cis‐10‐Pentadecenoic; C16:0 = Palmitic; C16:1 = Palmitoleic; C17:0 = Heptadecenoic; C17:1 = cis‐10‐Heptadecenoic; C18:0 = Stearic; C18:1n9t = Elaidic; C18:1n9c = Oleic; C18:2n6t = Linolelaidic; C18:2n6c = Linoleic; C20:0 = Arachidic; C20:1c1n9 = cis‐11‐Eicosenoic; C18:3n3 = Linolenic; C20:2c11 = cis‐11,14‐Eicosadienoic; C20:4n6 = Arachidonic; C22:2 = cis‐13,16‐Docosadienoic; C24:0 = Lignoceric) were identified in the study.

Oil contents and fatty acid compositions of the seed oil (Table 3) varied depending on the genotypes and cultivars in this study. Linoleic (42.01%), palmitic (31.65%), oleic (18.39%), and stearic acids (3.20%) consisted of the largest fraction of fatty acids, on average. Linoleic acid percentages ranged between 35.09% and 49.73%. GAN‐21 (49.73%) and MGL‐9 (46.14%) had the highest percentages of linoleic acid. AYD‐11, with relatively high seed oil content (18.16%), had also a high linoleic acid content (44.61%). Linoleic acid content in our samples was similar to the value (38.98%) reported for okra (Gemede et al. 2015). Marmara 1, AYD‐11, YLV‐22, YLV‐23, MGL‐14, AYD‐18, and AYD‐12 had relatively high linoleic acid content (35.09%–49.73%) and low palmitic acid content (18.01%–42.05%). Palmitic acid ranged between 18.01% and 42.05%, with an average of 31.65%. The highest palmitic acid percentage was detected in MGL‐8 (42.05%), where the lowest content of palmitic acid (18.01%) was determined in AYD‐18. Oleic acid percentages ranged between 12.53% and 22.45%. GAN‐21 (22.45%) and STD‐20 (21.71%) had the highest percentages of oleic acid content. Stearic acid values varied from 0.90% to a maximum of 4.02% (Marmara 1). The fraction of other minor fatty acids reached 2.06% (eicosadienoic acid), followed by docosadienoic acid (1.95%), eicosenoic acid (1.57%), arachidic acid (1.24%), heptadecenoic acid (1.18%), lignoceric acid (1.17%), palmitoleic acid (1.04%), and linolelaidic acid (1.0%).

**TABLE 3:** Oil contents and ratio of fatty acids of okra landraces and cultivars.

Genotype name	Oil content of seed (%)	C_14:0_	C_15:0_	C_15:1_	C_16:0_	C_16:1_	C_17:0_	C_17:1_	C_18:0_	C_18:1n9t_	C_18:1n9c_	C_18:2n6t_	C_18:2n6c_	C_20:0_	C_20:1 c1n9_	C_18:3n3_	C_20:2 c11_	C_20:4n6_	C_22:2_	C_24:0_
BLK‐1	14.97	0.28	0.55	—	29.87	1.04	0.18	0.43	3.02	0.14	16.11	0.64	42.07	0.30	—	0.88	1.31	0.62	1.18	0.71
MGL‐2	14.17	—	0.68	—	27.94	0.91	0.13	0.28	2.90	0.08	17.93	0.77	42.11	0.56	—	0.69	1.50	0.68	1.95	0.71
MGL‐3	12.55	0.24	0.03	—	31.36	0.34	0.15	0.16	3.76	0.15	17.77	0.56	44.01	0.46	0.20	0.02	0.27	—	—	0.13
MGL‐4	14.40	0.34	—	—	29.47	0.30	0.13	0.83	3.14	—	20.41	0.41	43.59	0.49	—	0.15	0.21	0.04	—	0.19
MGL‐5	13.96	0.52	0.04	0.03	39.76	0.53	0.13	0.04	2.90	0.16	15.60	0.04	38.37	0.27	0.16	0.14	0.19	0.76	0.10	0.05
MGL‐6	13.77	0.22	0.03	0.02	31.34	0.37	0.16	0.19	3.39	0.14	17.44	0.58	44.76	0.37	0.21	—	0.20	0.04	—	0.08
MGL‐7	12.92	0.35	—	0.24	32.40	0.68	0.16	0.19	3.32	0.23	13.11	0.79	43.29	0.44	0.25	0.47	1.20	0.45	0.91	0.76
MGL‐8	14.20	0.26	—	—	42.05	0.59	0.12	0.15	2.84	—	12.53	1.00	35.09	0.40	—	0.39	1.31	0.39	1.11	1.17
MGL‐9	13.58	0.26	—	—	30.19	0.53	0.14	0.15	2.65	0.23	17.48	0.67	46.14	0.44	—	0.16	0.42	0.25	—	0.06
MGL‐10	14.50	0.15	0.02	—	28.17	0.38	0.10	0.19	3.73	0.18	21.23	0.56	44.11	0.37	0.21	0.01	0.18	0.04	0.02	0.09
AYD‐11	18.16	0.17	0.03	0.02	29.75	0.39	0.14	0.06	3.48	0.16	19.26	0.61	44.61	0.40	0.18	—	0.21	0.05	—	0.09
AYD‐12	17.77	0.44	—	—	35.74	0.54	0.13	0.14	3.07	0.30	18.55	0.68	36.96	0.36	0.60	0.25	0.17	0.17	0.33	0.25
AYD‐13	13.94	0.22	0.03	—	30.89	0.40	0.12	0.18	3.27	0.14	18.80	0.06	44.63	0.41	0.27	0.03	0.20	0.03	0.03	0.08
MGL‐14	16.52	0.40	—	0.57	34.24	0.65	—	—	0.90	0.32	14.19	0.60	37.63	1.24	—	0.96	2.06	0.58	1.21	0.41
UIS‐15	15.36	0.35	0.02	0.08	28.01	0.62	0.19	0.12	3.78	0.13	20.14	0.37	42.89	0.54	0.16	0.02	0.52	0.50	0.36	0.51
UIS‐16	15.56	0.38	—	0.07	36.11	0.58	0.15	0.14	3.14	0.11	18.25	0.39	37.58	0.51	0.16	0.22	0.73	0.44	0.27	0.11
USK‐17	14.71	0.22	0.02	0.02	31.14	0.35	0.12	0.16	3.71	0.16	20.42	0.54	41.71	0.43	0.19	0.09	0.23	0.05	0.29	0.11
AYD‐18	18.83	0.18	0.02	0.01	31.12	0.38	0.10	0.16	3.38	0.13	19.82	0.57	43.05	0.40	0.22	0.01	0.21	0.06	0.02	0.09
GAN‐19	12.15	0.47	0.04	—	36.63	0.48	0.14	0.17	2.96	0.16	17.64	0.60	39.36	0.32	0.31	0.05	0.17	0.23	0.07	0.08
STD‐20	13.97	0.19	0.02	0.02	29.59	0.31	0.09	0.05	3.81	0.14	21.71	0.50	42.23	0.43	0.22	0.01	0.23	0.06	0.01	0.10
GAN‐21	12.59	0.46	—	—	18.01	0.29	0.10	0.09	3.55	0.01	22.45	0.29	49.73	0.35	1.57	0.87	0.15	0.74	1.12	0.13
YLV‐22	16.42	0.84	0.02	0.01	29.11	0.46	0.34	0.17	3.28	0.16	19.42	0.63	43.23	0.44	0.33	0.05	0.22	0.21	0.12	0.12
YLV‐23	17.02	0.54	0.20	0.02	30.86	0.46	0.13	0.19	3.13	0.15	19.54	0.59	43.03	0.34	0.20	—	0.17	0.03	—	0.08
Akköy‐41	15.42	0.31	—	—	33.88	0.42	0.10	0.20	3.00	0.15	19.17	0.54	41.49	0.34	0.18	—	0.17	—	—	0.08
Kabaklı‐11	14.68	0.39	—	—	36.89	0.51	0.09	0.17	2.97	0.15	19.24	0.52	38.32	0.27	0.17	0.08	0.13	—	0.10	—
Marmara 1	16.29	0.20	0.07	—	28.35	0.49	1.18	—	4.02	—	19.82	0.75	42.35	0.55	—	0.39	0.31	—	1.14	0.17
Mean	14.94	0.34	0.11	0.09	31.65	0.50	0.18	0.19	3.20	0.16	18.39	0.55	42.01	0.44	0.30	0.27	0.49	0.29	0.54	0.25

*Note:* C_14:0_ = Miristic; C_15:0_ = Pentadecanoic; C_15:1_ = cis‐10‐Pentadecanoic; C_16:0_ = Palmitic; C_16:1_ = Palmitoleic; C_17:0_ = Heptadecanoic; C_17:1_ = cis‐10‐Heptadecanoic; C_18:0_ = Stearic; C_18:1n9t_ = Elaidic; C_18:1n9c_ = Oleic; C_18:2n6t_ = Linolelaidic; C_18:2n6c_ = Linoleic; C_20:0_ = Arachidic; C_20:1c1n9_ = cis‐11‐Eicosenoic; C_18:3n3_ = Linolenic; C_20:2c11_ = cis‐11,14‐Eicosadienoic; C_20:4n6_ = Arachidonic; C_22:2_ = cis‐13,16‐Docosadienoic; C_24:0_ = Lignoceric.

There was a 1.5–3.7‐fold variation between minimum and maximum values for palmitic, stearic, oleic, and linoleic acid percentages within the genotypes that can be exploited in breeding programs. In another study (Gemede et al. 2015), the fatty acid composition of okra seeds ranged between 13.24% and 22.26% for palmitic acid, 2.26%–10.93% for stearic acid, 13.90%–24.07% for oleic acid, and 35.31%–43.93% for linoleic acid. (Moosavi et al. 2018) showed that the most abundant fatty acid was linoleic acid (38%–40%), palmitic acid (29%–30%), and oleic acid (19%–22%) in 3 Iranian okra genotypes. (Anwar et al. 2010) reported that linoleic (30.31%), palmitic (30.23%), oleic (29.09%), and stearic (4.93%) acids are the most important fatty acids. Omoniyi, Idowu, and Adeola (2018) reviewed current literature on domestic and industrial use of okra seed and reported that seed oils contained commonly 21.97%–30.42% palmitic, 36.56%–49.54% linoleic, 16.81%–27.49% oleic, and 2.64%–4.75% stearic acids, but in small amounts of 0.19%–0.38% myristic, 0.31%–0.98% palmitoleic, 0.17%–2.64% linolenic, and 0.16%–0.52% behenic acids.

Okra oil had certain similarities to cotton seed oil. Linoleic acid (32.22%–43.07%), linolenic acid (6.79%–12.34%), and oleic acid (4.31%–6.98%) were the most common fatty acids in cotton seed oil (Liu et al. 2019). Okra oleic acid content in our experiment (18.39%) was comparable with corn (24.8%), soybean (23.2%), and sunflower seed oil (17.7%) (Gemede et al. 2015).

Saturated fatty acids (SFA) in the study ranged between 22.60/100 g (GAN‐21) and 46.84/100 g (MGL‐8). The highest value of SFA/UFA (0.89) was also found in MGL‐8. Levels of monounsaturated fatty acids (MUFA) and polyunsaturated fatty acids (PUFA) were the highest (24.41/100 g and 52.90/100 g, respectively) in GAN‐21.

### 3.3 Gossypol Contents of Plant Fractions

Gossypol contents in fruit, oil cake, and cold‐pressed oil ranged between < LOQ‐2.116, < LOQ‐7.008, and < LOQ‐62.459 mg/kg, respectively (Table 4). Determined gossypol concentrations in okra fractions were far below the limits set by the FDA in cottonseed (450 mg/kg) (FDA 2020). Previous studies reported that okra seed contained 0–29 mg/kg of gossypol (Martin and Rhodes 1983). This difference can be attributed to seasonal, locational, and genotypic variations in okra.

**TABLE 4:** Gossypol contents (mg/kg) in fruit, oil cake, and oil of okra genotypes and cultivars investigated.

Genotype name	Fruit	Oil cake	Oil
BLK‐1	1.716	—	< LOQ
MGL‐2	< LOQ	< LOQ	< LOQ
MGL‐3	0.198	< LOQ	12.610
MGL‐4	0.082	< LOQ	8.980
MGL‐5	0.465	< LOQ	< LOQ
MGL‐6	< LOQ	< LOQ	1.138
MGL‐7	0.745	< LOQ	1.894
MGL‐8	0.269	< LOQ	0.390
MGL‐9	< LOQ	3.564	< LOQ
MGL‐10	< LOQ	< LOQ	0.697
AYD‐11	< LOQ	< LOQ	19.857
AYD‐12	0.321	< LOQ	0.772
AYD‐13	0.565	0.469	62.459
MGL‐14	< LOQ	0.022	< LOQ
UIS‐15	2.116	1.483	< LOQ
UIS‐16	0.830	0.896	0.857
USK‐17	0.187	< LOQ	< LOQ
AYD‐18	0.282	< LOQ	0.267
GAN‐19	< LOQ	0.553	< LOQ
STD‐20	0.522	7.008	< LOQ
GAN‐21	1.724	4.098	2.929
YLV‐22	< LOQ	0.024	< LOQ
YLV‐23	< LOQ	< LOQ	0.654
Akköy‐41	0.364	< LOQ	1.411
Kabaklı‐11	0.567	0.758	1.257
Marmara 1	0.763	< LOQ	< LOQ
Min	< LOQ	< LOQ	< LOQ
Max	2.116	7.008	62.459
Mean	0.451	0.726	4.468
SE	0.115	0.326	2.492
SD	0.584	1.661	12.705

Gossypol is toxic to non‐ruminant animals, restricting the use of cottonseed meal as feedstuff (Blom et al. 2001; Henry, Pesti, and Brown 2001). Cotton breeders developed low or zero (glandless) gossypol cotton cultivars (Cai, Xie, and Liu 2010). However, gossypol and related terpenoids protect the plants from pests and some diseases (Kong, Daud, and Zhu 2010). The presence of gossypol in plant leaves, stems, and roots is hence desirable since they confer natural resistance against pests and diseases (Hagenbucher et al. 2019). Dry leaves of conventional glanded cotton (*Gossypium hirsutum*) contained as much as 8470 mg/kg gossypol (Sotelo et al. 2005). *Gossypium barbadense* seed contained up to 3400 mg/kg gossypol (Percy, Calhoun, and Kim 1996). Glandless cotton had 120 mg/kg seed gossypol content, while glanded cotton had 9000 mg/kg (Stipanovic et al. 1984). Okra leaf samples in the study had negligible amounts of gossypol (LOQ‐62.459 mg/kg, unpresented data) when compared with far higher amounts reported for cotton. The directive of the European Union (2002L0032 ‐ EN ‐ 26.02.2013 ‐ 017.001) states that the maximum free gossypol concentrations for cottonseed are 5000 ppm and 1200 ppm for cottonseed meal or cake (EU 2002a). In the study, okra landraces, lines, and cultivars had almost zero gossypol in oil cake, which may serve as virtually gossypol‐free food and feedstuff. Leaf gossypol content was correlated with fruit gossypol content (*r* = 0.718**, *p* < 0.01) but not with gossypol content in oil cake and oil fraction. This may indicate that gossypol synthesized in leaves was not translocated to seeds.

### 3.4 PCA and Proximity Cluster Dendrogram by Oil Composition

The data matrix of oil content and 19 determined fatty acids in the cold‐pressed okra seed oil was subjected to PCA to reduce the descriptor associated with the data set. PCA on oil composition values determined 6 components having eigenvalues (1.268–7.845) explaining 83.842% of total variance (Table 5). The first 2 variances accounted for 32.687% and 21.476% of variance, respectively (54.164% in total). Of the fatty acids analyzed, cis_11,14_eicosadienoic acid (0.967), palmitoleic acid (0.808), lignoceric acid (0.775), cis_13,16_docosadienoic acid (0.768), and linolenic acid (0.756) significantly and positively contributed to the first component, whereas oleic acid (−0.781) and stearic acid (−0.687) negatively contributed. Elaidic acid (−0.636) and pentadecenoic acid (0.599) were the most important fatty acids contributing to the second component.

**TABLE 5:** Principle component analysis of seed oil content. fatty acid contents, and gossypol content of fruit, oil cake, and oil in okra lines investigated.

	Components with eigenvalues > 1								
	1	2	3	4	5	6	7	8	9
Eigenvalue	8.02	4.89	2.80	2.08	1.74	1.40	1.19	1.08	1.03
Variance (%)	29.71	18.10	10.37	7.69	6.46	5.17	4.41	3.98	3.80
Cumulative variance (%)	29.71	47.81	58.18	65.87	72.34	77.51	81.92	85.90	89.71
Oil	0.051	−0.203	0.065	0.465	0.385	0.485	0.287	−0.186	0.061
Palmitic acid	0.672	−0.597	−0.237	−0.184	−0.092	−0.019	0.035	0.245	0.122
Stearic acid	−0.839	0.323	−0.249	−0.069	0.206	0.002	0.075	−0.035	0.103
Oleic acid	−0.738	0.177	0.138	0.246	−0.182	0.432	−0.055	0.103	0.295
Linoleic acid	−0.449	0.711	0.223	0.288	−0.139	−0.131	0.002	0.098	−0.288
Fruit gossypol	−0.125	0.561	−0.051	−0.516	0.121	0.076	0.307	−0.102	0.212
Oil cake gossypol	−0.372	0.240	0.276	−0.311	0.093	−0.188	−0.464	−0.089	0.416
Oil gossypol	−0.242	−0.102	0.092	0.105	−0.443	−0.476	0.568	−0.120	−0.004
Miristic acid	0.026	−0.205	0.363	−0.425	0.141	0.468	0.146	0.296	−0.399
Pentadecanoic acid	0.248	0.578	−0.423	0.206	−0.266	0.272	−0.005	−0.357	−0.047
cis‐10‐Pentadecanoic	0.613	0.130	0.661	0.203	0.106	−0.100	0.076	0.132	0.082
Palmitic acid	0.625	−0.708	−0.165	−0.235	−0.021	0.001	0.045	−0.053	0.085
Palmitoleic acid	0.701	0.447	−0.257	−0.027	−0.082	0.226	0.101	−0.333	−0.058
Heptadecanoic acid	−0.140	0.150	−0.391	0.190	0.643	−0.121	0.359	0.214	−0.095
cis‐10‐Heptadecanoic acid	−0.067	0.084	−0.350	0.191	−0.683	0.322	−0.034	0.429	−0.003
Stearic acid	−0.742	0.042	−0.489	−0.102	0.205	−0.150	0.018	−0.097	−0.024
Elaidic acid	0.317	−0.281	0.595	0.161	−0.005	0.079	−0.130	−0.482	−0.239
Oleic acid	−0.888	0.087	0.061	0.151	0.091	0.266	−0.038	−0.015	0.251
Linolelaidic acid	0.452	0.069	−0.395	0.343	0.360	−0.036	−0.494	0.092	−0.233
Linoleic acid	−0.698	0.458	0.139	0.196	−0.169	−0.235	−0.071	0.012	−0.299
Arachidic acid	0.485	0.226	0.525	0.448	0.163	−0.100	0.111	0.230	0.296
cis‐11‐Eicosenoic acid	−0.472	0.330	0.368	−0.479	0.109	0.056	−0.074	0.094	−0.231
Linolenic acid	0.516	0.757	0.149	−0.071	0.030	0.052	0.031	0.144	−0.010
cis‐11,14‐Eicosadienoic	0.854	0.447	0.058	0.138	−0.053	−0.078	−0.025	0.014	0.120
Arachidonic acid	0.469	0.607	0.170	−0.478	−0.073	0.153	0.066	−0.063	0.024
cis‐13,16‐Docosadienoic	0.533	0.757	−0.166	0.012	0.164	−0.036	0.035	0.077	0.034
Lignoceric acid	0.688	0.402	−0.376	−0.115	−0.033	−0.124	−0.073	0.079	0.019

**FIGURE 4:**
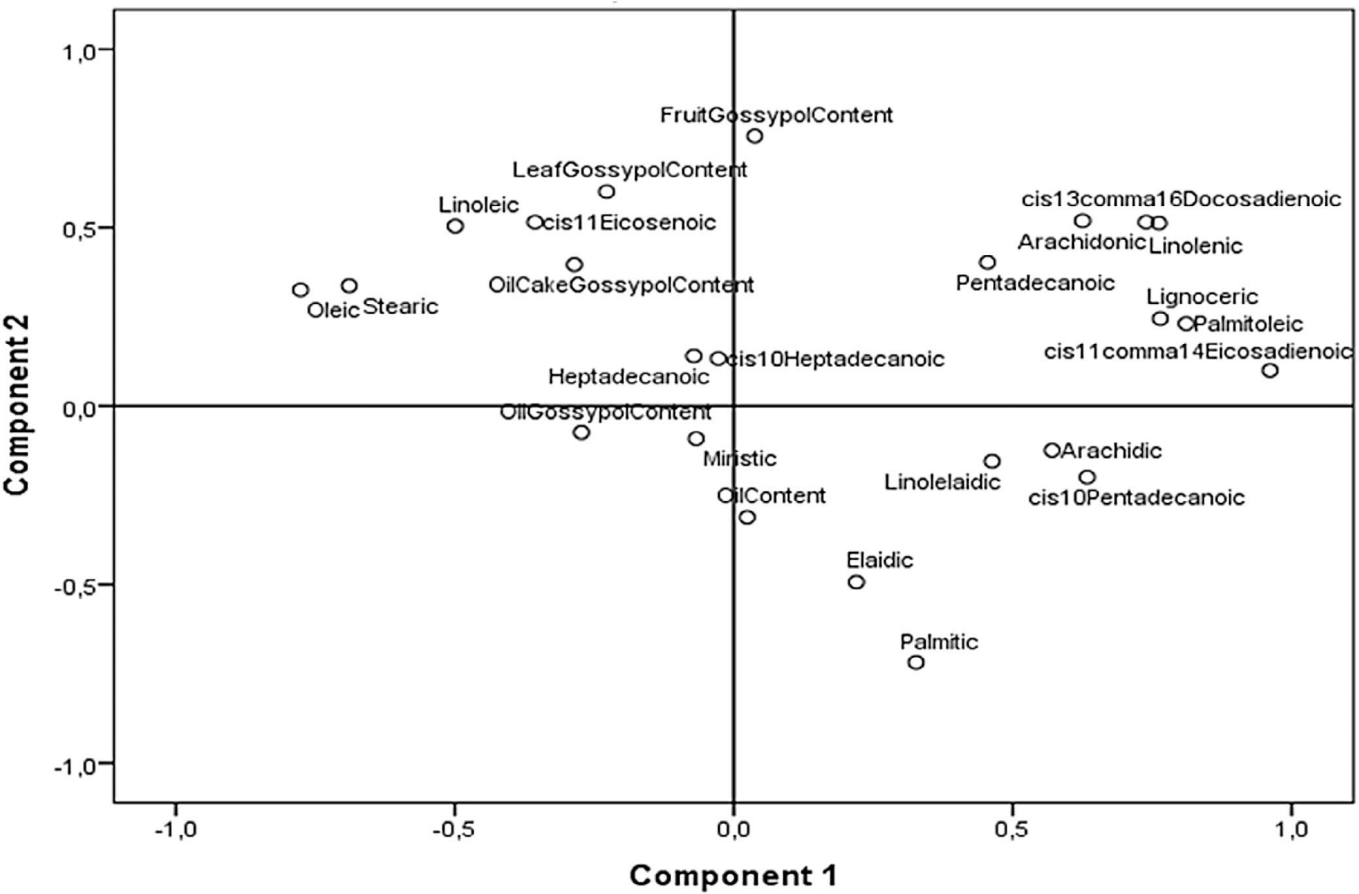
Bi‐plot of oil contet and fatty acid compostion of okra genotypes and cultivars.

**FIGURE 5:**
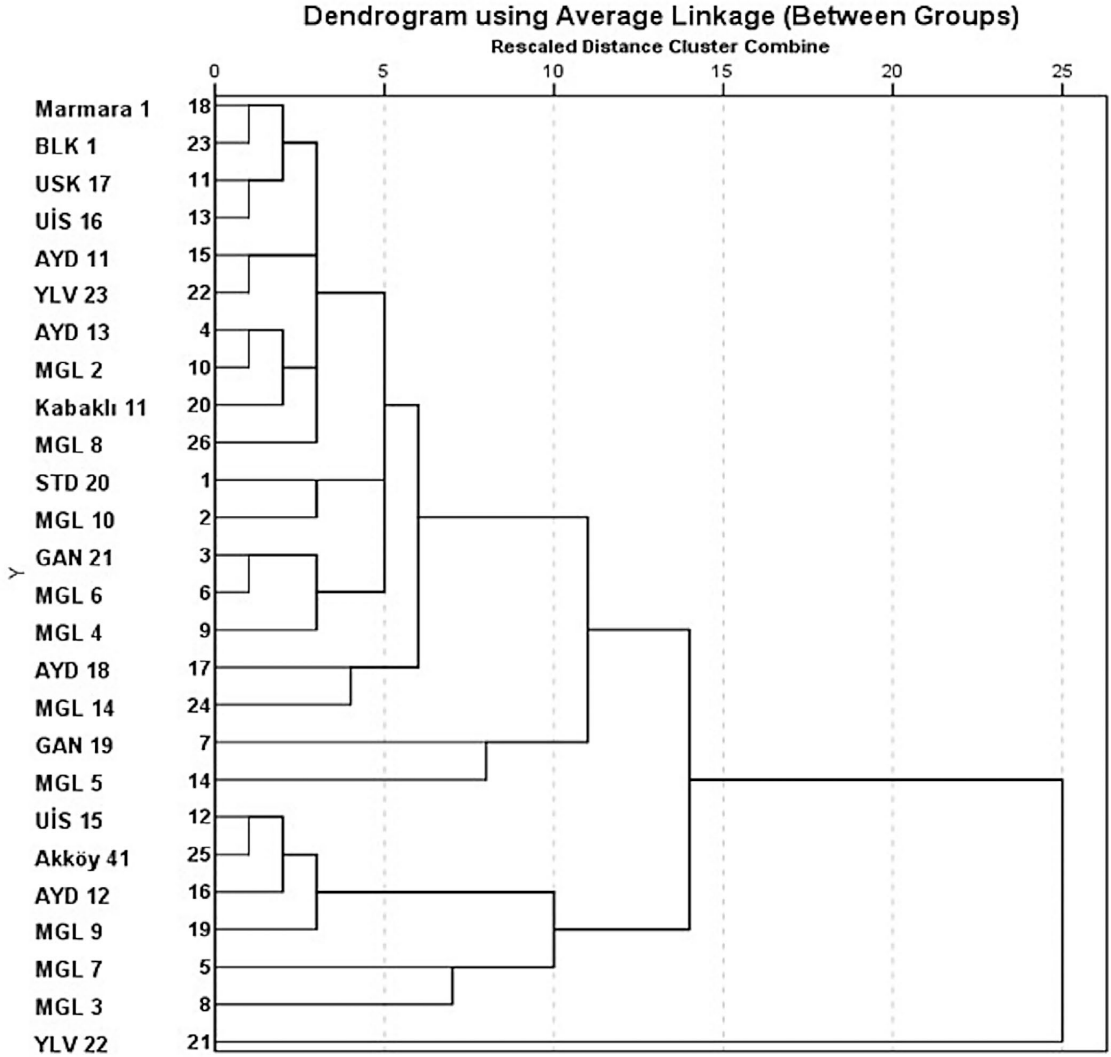
Dendogram of proximity of okra genotypes and cultivars by oil content and fatty acid composition.

A biplot graph positioned oil content away from major fatty acids such as linolenic and palmitic acids but close to oleic acid (Figure 4). Oil content was significantly correlated with only oleic acid content (*r* = 0.595, *p* < 0.001) and with MUFA in general (*r* = 0.604, *p* < 0.001) (data not presented). Proximaty dendogram produced separated three groups of genotypes (Figure 5). YLV‐22 stands out as a separate line (Group I), with MGL‐3, MGL‐7, MGL‐9, AYD‐12, Akköy‐11, and UIS‐15 forming Group 2, while the rest of the genotypes including MGL‐5 and Marmara‐1 forming the 3rd Group. Marmara‐11 and BLK‐1 are the most distant cultivars to MGL‐3 and YLV‐22. The data presented here may be used in breeding high‐oil content cultivars and custom‐specific fatty acid lines. The highest variations were calculated for palmitic acid (21.496), linoleic acid (10.456), and oleic acid (6.054) contents, which can be exploited in breeding programs.

## 4 Conclusion

This study demonstrated that some okra genotypes had up to 18.83% seed oil contents. Seed oils comprised also high‐quality linoleic (42.01%), palmitic (31.65%), oleic (18.39%), and stearic (3.20%) fatty acids. Fruit, oil cake, and seed oil gossypol contents (< LOQ‐2.116, < LOQ‐7.008 and < LOQ‐62.459 mg/kg) were far below the international limits. The variation in oil content and oil composition in the study can be exploited in order to develop oil seed type okra cultivars. High oil cultivars may enhance the prospect of okra as an oil crop. In conclusion, the study obviously indicates that the combination of experimental fatty acid information along with a chemometric analysis (PCA) can be successfully employed by okra researchers to give more information on variation in okra cultivars than is only with the experimental data alone.

Twenty‐six okra (*A. esculentus*) lines, harvested from some locations in Turkiye, were examined in the present study, and in the future, more lines and some other species (i.e., *A. crinitis*, *A. ficulneus*, and *A. tuberculatus*) collected from large areas should be studied for the determination of oil contents, fatty acid profiles, and also gossypol contents.

## Author Contributions

**Faik Kantar:** conceptualization (equal), data curation (equal), writing – review and editing (equal). **Mehmet Fatih Cengiz:** formal analysis (equal), investigation (equal), writing – review and editing (equal). **Sabri Erbaş:** formal analysis (equal). **Ümit Babacan:** formal analysis (equal).

## Consent

All authors agreed to publish the manuscript.

## Conflicts of Interest

The authors declare no conflicts of interest.

## Data Availability

The datasets analyzed during the current study are available from the corresponding author upon reasonable request.
